# Kawasaki disease: abnormal initial echocardiogram is associated with resistance to IV Ig and development of coronary artery lesions

**DOI:** 10.1186/s12969-018-0264-7

**Published:** 2018-07-18

**Authors:** Dima Chbeir, Jean Gaschignard, Ronan Bonnefoy, Constance Beyler, Isabelle Melki, Albert Faye, Ulrich Meinzer

**Affiliations:** 1 Service de pédiatrie générale, maladies infectieuses et médecine interne, Centre de référence des rhumatismes inflammatoires et maladies auto-immunes systémiques rares de l’enfant (RAISE), Hôpital Robert Debré, Assistance Publique Hôpitaux de Paris, 75019 Paris, France; 2 Centre de recherche sur l’inflammation, Institut National de la Santé et de la Recherche Médicale, Université Paris Diderot-Sorbonne Paris-Cité, UMR 1149, 75018 Paris, France; 3 Service de cardiologie pédiatrique, Hôpital Robert Debré, Assistance Publique-Hôpitaux de Paris, 75019 Paris, France; 4 Institut Pasteur, Unité biologie et génétique de la paroi bactérienne, 75015 Paris, France

**Keywords:** Kawasaki disease, Vasculitis, Pediatric Rheumatology, coronary artery

## Abstract

**Background:**

Kawasaki disease (KD) is an acute febrile systemic vasculitis that affects small and medium blood vessels. Intensified treatments for the most severely affected patients have been proposed recently, and the early identification of KD patients at high risk for coronary artery aneurysms (CAA) is crucial. However, the risk scoring systems developed in Japan have not been validated in European populations, and little data is available concerning the link between initial echocardiogram findings other than high z-scores and cardiac prognosis.

**Methods:**

In order to investigate whether the presence of any abnormalities, other than high z-scores in first echocardiogram, are associated with resistance to IV immunoglobulins and/or subsequent development of CAA, we retrospectively analyzed data from children diagnosed with KD between 2006 and 2016 at a tertiary Hospital in Paris, France.

**Results:**

A total of 157 children were included. The initial echocardiogram was performed after a median of 7 days of fever and was abnormal in 48 cases (31%). The initial presence of any echocardiographic abnormality (coronary artery dilatation, CAA, pericardial effusion, perivascular brightness of the coronary arteries, left-ventricular dysfunction and mitral insufficiency) was strongly associated with resistance to intravenous immunoglobulin (*p* = 0.005) and development of coronary artery lesions within the first 6 weeks of disease (*p* = 0.01). All patients (*n* = 7) with persistent coronary abnormalities at 1 year already had an abnormal initial echocardiogram. Severity scoring systems from Japan had low sensitivity (0–33%) and low specificity (71–82%) for predicting immunoglobulin resistance or cardiac involvement.

**Conclusions:**

In European populations with mixed ethnic backgrounds, the presence of any abnormalities at the initial echocardiogram may contribute to early identification of patients with severe disease.

## Background

Kawasaki disease (KD) is an acute febrile systemic vasculitis that affects blood vessels of small and medium caliber, with a tropism for the coronary arteries [1, 2]. It is a leading cause of acquired heart disease in industrialized countries [3, 4], and the etiology remains largely unknown [2]. No specific test are available to confirm the diagnosis. The diagnosis of KD is based on internationally accepted clinical criteria [1, 5], combining fever lasting ≥ 5 days, with at least four of the following clinical signs: bilateral conjunctival injection, changes affecting the lips and oral cavity (injection of oral and pharyngeal mucosa), polymorphous exanthema, changes to the peripheral extremities, and cervical lymphadenopathy ≥15 mm.

Cardiac involvement affects the prognosis of children with coronary artery aneurysms (CAA), occurring in 25–30% of untreated KD patients [1, 6, 7]. The cardiac prognosis has been dramatically improved by intravenous injection of 2 g/kg of human immunoglobulin (IV Ig) [8, 9]. However, 11 to 28% of patients are resistant to IV Ig and show a higher rate of coronary disease [10–13]. Recent studies suggest that the combination of IV Ig with corticosteroids for the initial treatment may be more efficient than IV Ig alone to prevent cardiac complications in severe disease [14–16]. Other treatment approaches for patients with the most severe disease, including interleukin-1 and tumor necrosis factor alpha targeting drugs are the subject of ongoing debate [1, 2, 16, 17]. In order to adequately implement such intensified treatment approaches, it is necessary to establish markers that allow early identification of patients at high risk for CAA.

Several scores have been developed to identify patients at high risk of resistance to IV Ig or coronary disease [17–19]. They have essentially been validated in Japan [21, 22], but have shown low sensitivity to predict IV Ig resistance in other populations, including European populations [20, 22–25]. Echocardiography is the imaging modality of choice for detection of coronary artery abnormalities and assessment of myocardial function. It is also useful for risk stratification of patients with KD. Recent studies showed that baseline zMax ≥2.0 in children with Kawasaki disease is predictive for later development of CAA [26]. However, little data is available on the value of other abnormalities (pericardial effusion, perivascular brightness of the coronary arteries, left-ventricular dysfunction and mitral insufficiency), recorded during the first diagnostic echocardiogram, to predict secondary CAA [27].

In this retrospective observational study we aim to determine whether the presence of any abnormality at initial echocardiogram is predictive for CAA and/or resistance to conventional IV Ig treatment, and to study the validity of Japanese clinical scoring systems in European patients with a mixed genetic background.

## Methods

We included all children aged < 18 years diagnosed with KD and hospitalized between January 2006 and December 2016 at the Robert-Debré University Hospital in Paris, France. This hospital predominantly receives patients form the North-Western region of Paris and suburbs. For each patient, we collected demographic, clinical, biological, echocardiographic and therapeutic data. KD was assessed based on internationally accepted clinical criteria [1, 5]. Patients for which another diagnosis was confirmed during the follow-up were excluded.

All echocardiograms were reviewed by two different cardiologists. Coronary dilatations were defined according to the Japanese Ministry of Health, and aneurysm according to the American Heart Association [1, 28]. Resistance to treatment was defined as the persistence of fever (temperature > 38.0 °C) 48 h after the IV Ig infusion. ∆CRP was defined as the difference (%) between the CRP measured 48 h after the IV Ig infusion and CRP measured before Ig infusion.

Severity scores were calculated according to published definitions [17–19]. For Kobayashi: Alanine aminotransferase (ALT) ≥ 100 IU/l (2 points), sodium ≤133 mmol/l (2 points), disease duration ≤4 days (2 points), neutrophil percentage ≥ 80% (2 points), C Reactive Protein (CRP) ≥ 100 mg/l (1 point), age ≤ 12 months (1 point) and platelet count ≤300,000/mm3 (1 point). For Egami: disease duration ≤4 days (1 point), ALT ≥80 IU/l (2 points), platelet count ≤300,000/mm3 (1 point), CRP ≥ 80 mg/l (1 point) and age < 6 months (1 point). For Sano: ALT ≥200 IU/l (1 point), bilirubinemia ≥0.9 mg/dl (1 point), CRP ≥ 70 mg/l (1 point). Patients with scores ≥5 (Kobayashi), ≥ 3 (Egami) or ≥ 2 (Sano) were considered as severe.

Results were expressed as numbers and percentages for categorical variables, and median (with interquartile range) or mean (with 95% confidence interval) for quantitative ones. Comparisons were performed using the Fisher’s exact test for categorical variables and the Mann-Whitney U test for quantitative variables. A two-sided *p*-value < 0.05 was considered statistically significant.

All data were collected anonymously. This study has been approved by French data protection authorities (Commission Nationale Informatique et Libertés, No 2014908).

## Results

Between January 2006 and December 2016, 166 children aged < 18 years were hospitalized at the Robert Debré University Hospital and initially diagnosed with KD; 157 children were included in this study. Nine children were excluded because a diagnosis other than KD was established during follow up: seven had a viral infection, one a systemic juvenile idiopathic arthritis and one a vasculitis other than KD.

### Patients’ characteristics and initial management

Age at diagnosis ranged from 1 month to 12 years (median 25 months, IQR = 13–43); 85% were less than 5 years old. Sex ratio was 1.4 (92 boys and 65 girls). The ethnic backgrounds of patients were: Sub-Saharan African (32%), European (27%), Northern African (21%), Asian (15%), Caribbean (3%) and Middle Eastern (2%).

Sixty patients had complete Kawasaki disease, while 72 had three major signs in addition to fever. Patients with incomplete KD had at least two minor signs and/or a cardiac involvement: 18 patients had two signs, six had one sign, and one had no signs of KD, but had a CAA. All children had a fever lasting for more than 5 days. Among the 25 patients that had less than three major signs, 4 patients showed dilatation of the coronary arteries and 5 patients a CAA.

The major clinical signs found were polymorphous exanthema (83%), changes to the lips and oral cavity (injection of oral and pharyngeal mucosa) (78%), conjunctivitis (73%), changes to the peripheral extremities or perineal area (44%) and lymphadenopathy ≥15 mm (41%). Patients with complete and incomplete KD had a similar sex-ratio (respectively 1.4 and 1.5, *p* = 0.88) and similar median age (respectively 27 and 22 months).

All children had received IV Ig 2 g/kg. The median time between the beginning of the fever and the first IV Ig dose was 6 days (IQR = 5–7). Due to initial cardiac abnormalities, 4 patients received corticosteroids along with the first dose of IV Ig (2 mg/kg/day for 2–5 days before switch to oral administration and subsequent tapering. One child with coronary aneurysms received Infliximab 5 days after the second dose of IV Ig because of persistent elevated CRP. All patients also received acetylsalicylic acid (initial dose 50–100 mg/kg/day).

### Abnormalities at the initial echocardiogram

Patients had an initial echocardiogram after a median of 7 days of fever (IQR 6–9). At least one abnormality was present in 48/157 children (31%): 24 had a mild coronary arterial dilatation, 15 a pericardial effusion, seven a perivascular brightness of the coronary arteries, seven a CAA (three small, three medium and one large), six a left-ventricular dysfunction, and two a mitral insufficiency (Table 1). Perivascular brightness included the left coronary artery in four cases and was diffuse in three cases. Perivascular brightness of the coronary arteries was the only abnormality in five cases. In two patients it was associated with a mild pericardial effusion. The ratio of abnormal initial echocardiogram findings was similar in patients with complete and incomplete KD (respectively 16/60 and 32/97, *p* = 0.48).

**Table 1:** Patients’ characteristics according to the response to immunoglobulin infusion (*N* = 157)

	Resistant (*N* = 45) N (%)	Responder (*N* = 112) N (%)	*p*
Demographics			
Sex (M/F)	24/21	68/44	.36
Age	31 [24–40]	31 [27–36]	.96
< 12 months	13 (29)	22 (20)	.39
12–23 months	7 (16)	34 (30)	.10
≥ 24 months	25 (55)	56 (50)	.47
Clinics			
Fever length before Ig (days)	7 [5–8]	7 [6–8]	.33
Lymphadenopathy	18 (40)	47 (42)	.72
Rash	39 (87)	91 (81)	.63
Conjunctivitis	39 (87)	76 (68)	**.03**
Enanthem	33 (73)	89 (79)	.67
Modification of the extremities	18 (40)	51 (46)	.47
Fever length after Ig (hours)	60 [56–64]	24 [21–36]	**< 10** ^**−10**^
Biology			
Neutrophils (%)	70 [66–74]	65 [62–69]	.17
Platelet count (G/L)	434,900 [375,140-494,680]	392,000 [361,860-421,420]	.31
Hemoglobin (g/dl)	10.4 [9.6–10.4]	10.7 [10.4–10.9]	.60
CRP (mg/L)	182 [159–207]	145 [131–160]	**.01**
ΔCRP (%)	41 [30–52]	61 [56–66]	**.0004**
ASAT (UI/L)	46 [39–54]	60 [44–74]	.32
ALAT (UI/L)	55 [42–69]	76 [54–98]	.73
Bilirubin (μmol/L)	10 [6–14]	12 [8–15]	.91
LDH (UI/L)	781 [611–950]	1442 [1073-1810]	.62
Albumin (g/L)	23 [21–25]	26 [25–27]	.16
Sodium (mmol/L)	135 [134–136]	135 [134–136]	.44
Scores			
Kobayashi			1
< 5	32 (78)	86 (78)	
≥ 5	9 (22)	25 (22)	
Egami			.49
< 3	32 (76)	88 (82)	
≥ 3	10 (24)	19 (18)	
Sano			.50
< 2	22 (67)	66 (73)	
≥ 2	11 (33)	24 (27)	
Treatments			
1st dose IV Ig	45 (100)	112 (100)	1
2nd dose IV Ig	20 (40)	1	**< 10** ^**−8**^
Corticosteroids	17 (39)	2 (2)	**< 10** ^**−8**^
First echocardiogram results			
Normal	23 (51)	86 (77)	**.002**
Abnormal	22 (49)	26 (23)	**.002**
Coronary artery dilatation	11 (24)	13 (12)	**.05**
Pericardial effusion	7 (16)	8 (7)	.13
Perivascular brightness of the coronary arteries	4 (9)	3 (3)	.10
CAA	3 (7)	4 (4)	.40
Small (< 5 mm)	1 (2)	2 (2)	1
Median (5–8 mm)	1 (2)	2 (2)	1
Giant (> 8 mm)	1 (2)	0	.28
Left ventricular dysfunction	4 (9)	2 (2)	**.05**
Mitral insufficiency	1 (2)	1 (1)	.48
Abnormal late echocardiograms			
6 weeks	10 (22)	8 (7)	**.01**
Coronary artery dilatation	5	2	
Pericardial effusion	1	0	
Perivascular brightness of the coronary arteries	1	0	
CAA	3	6	
Small (< 5 mm)	0	4	
Median (5–8 mm)	2	2	
Giant (> 8 mm)	1	0	
Long-term	4 (9)	3 (3)	.10
Coronary artery dilatation	2	0	
CAA	2	3	
Small (< 5 mm)	1	3	
Median (5–8 mm)	1	0	
Giant (> 8 mm)	0	0	

Sole p-values < 0.05 are in boldfaced and p-values = 0.05 are not in boldfaced

The patient with a giant aneurysm on the initial echocardiogram was a 4 year-old boy from European background. The aneurysm was stable after 6 weeks. He initially presented adenopathy > 15 mm and edema of the hands and feet, no conjunctivitis and no cheilitis. CRP was 108 mg/L and fever lasted 72 h after Ig infusion. At the follow up visit, four years after KD onset, the aneurysm remained stable (10 mm) upon a dilatated coronary (5 mm); the electrocardiogram and holter were normal during rest and physical activity. The patient was treated with Coumadin and Aspirin.

Patients with abnormal echocardiographic findings received a second dose of IV Ig due to fever recurrence within 48 h more often than those with a normal echocardiogram (25% vs 8%, *p* = 0.009). They also more frequently received corticosteroids (27% vs 6%, *p* = 0.0003) or a combination of IV Ig and corticosteroids (15% vs 4%, *p* = 0.04).

### Initial abnormal echocardiographic findings predict cardiac evolution

A second echocardiogram was performed for all patients 6 weeks after initiation of treatment. It was abnormal for 18/157 patients (11%). Among these patients, 16/18 (89%) already had abnormalities at the initial echocardiogram. Both patients with abnormalities at 6 weeks, despite a normal initial echocardiogram, had a normal echocardiogram after 1 year. Data on echocardiogram after 1 year were available for 14/18 patients with abnormal echocardiogram at 6 weeks. Of these, seven patients had echocardiographic abnormalities after 1 year: five had a CAA (one medium, four small) and two a left coronary dilatation. The initial echocardiograms of the seven patients with abnormal echocardiogram after 1 year were performed after 5 to 19 days of fever; three had a CAA (two small, one medium), one a left coronary artery dilatation, one a pericardial effusion and two a left ventricular dysfunction.

All patients with abnormal cardiac findings at 1 year already had abnormalities at the first echocardiogram. The sensitivity of abnormal initial echocardiographic finding for the prediction of the development of secondary coronary artery lesion was 100%, while specificity was 73%. In the subgroup of patients who had an echocardiogram within the first 10 days of fever (*N* = 105), 37 had an abnormal initial echocardiogram and among these 5 developed secondary artery lesion. None of the 68 patients with normal initial echocardiogram developed secondary artery lesion. Thus, sensitivity and specificity were respectively 100 and 68% in this subgroup.

### Abnormal initial echocardiography is associated with resistance to IV Ig

Resistance to the first injection of Ig was observed in 45/157 patients (29%), for which fever above 38.0 °C persisted for a mean duration of 60 h after the end of Ig infusion. Sex and clinical presentation were comparable between the responder and the resistant groups (Table 1). Only conjunctivitis was more frequent among resistant children than responders (87% vs 68%, *p* = 0.03). Concerning biological features, only CRP before IV Ig and ∆CRP were significantly higher in resistant children compared to responders (183 vs 145 mg/l, *p* = 0.01 and − 41% vs − 61%, *p* = 0.0004, respectively) (Table 1).

Both dates for echocardiogram and Ig infusion were available for 117 patients. Echocardiogram was performed before Ig infusion in 50% of cases (*N* = 58), within 72 h after Ig infusion in 39% (*N* = 46), and after more than 3 days in 11% (*N* = 13).

The frequency of abnormal initial echocardiograms was significantly higher in resistant children compared to responders (49% vs 23%, *p* = 0.002): a significant difference was found for mild dilatation of the coronary arteries (24% vs 12%, *p* = 0.05) and left ventricular dysfunction (9% vs 2%, *p* = 0.05) and there was a trend for presence of CAA after 1 year (9% vs 3%, *p* = 0.10) (Table 1). Sensitivity of abnormal initial echocardiographic finding was 49% for the prediction of resistance to IV Ig, while specificity was 77%.

Similar results were observed when exclusively considering patients for whom the initial echocardiography was performed before IV Ig treatment: The frequency of abnormal initial echocardiograms was significantly higher for resistant children compared to responders (75% vs 40%, *p* = 0.04) (Table 2). In this group, sensitivity of abnormal initial echocardiographic finding was 75% for the prediction of resistance to IV Ig, while specificity was 60%.

**Table 2:** Characteristics of patients’ with initial echocardiogram prior to immunoglobulin infusion according to the response to immunoglobulin infusion (*N*=58)

	Resistant (*N* = 16) N (%)	Responder (*N* = 42) N (%)	*p*
Demographics			
Sex (M/F)	9/7	24/18	1
Age (mean)	33	29	0.32
< 12 months	8 (50)	11 (26)	0.12
12–23 months	1 (6)	10 (24)	0.26
≥ 24 months	7 (44)	21 (50)	0.77
Clinics			
Fever length before Ig (days)	6 [5–8]	7 [6–8]	.35
Lymphadenopathy	7 (44)	17 (40)	1
Rash	11 (69)	32 (76)	.74
Conjunctivitis	12 (75)	25 (60)	.37
Enanthem	8 (50)	30 (71)	.14
Modification of the extremities	5 (31)	22 (52)	.24
Fever length after Ig (hours)	62 [58–64]	24 [20–36]	< 10^−10^
Biology			
Platelets	463,500	405,900	.61
Neutrophils (%)	66	64	.47
Hemoglobin (g/dl)	10.3	10.3	.91
CRP (mg/L)	172	140	.23
ΔCRP (%)	48	62	.08
ASAT (UI/L)	46	61	.31
ALAT (UI/L)	49	81	.15
Bilirubin (μmol/L)	17	10	.54
LDH (UI/L)	855	636	.66
Albumin (g/L)	26	27	.96
Sodium (mmol/L)	136	136	.70
Scores			
Kobayashi			.66
< 5	10 (91)	29 (81)	
≥ 5	1 (9)	7 (19)	
Egami			1
< 3	9 (75)	26 (72)	
≥ 3	3 (25)	10 (28)	
Sano			.69
< 2	5 (63)	23 (70)	
≥ 2	3 (37)	10 (30)	
Treatments			
1st dose IV Ig	15 (100)	43 (100)	1
2nd dose IV Ig	5 (33)	0 (0)	**.0009**
Corticosteroids	5 (33)	1 (2)	**.004**
First echocardiogram results			
Normal	4 (25)	25 (60)	**.04**
Abnormal	12 (75)	17 (40)	**.04**
Coronary artery dilatation	4 (25)	5 (12)	.24
Pericardial effusion	3 (19)	6 (14)	.70
Perivascular brightness of the coronary arteries	1 (7)	2 (5)	.53
CAA	1 (7)	2 (5)	.53
Small (< 5 mm)	0	1	
Median (5–8 mm)	0	1	
Giant (> 8 mm)	1	0	
Left ventricular dysfunction	1	2	
Mitral insufficiency	0	0	
Abnormal late echocardiograms			
6 weeks	8 (50)	5 (12)	**.03**
Coronary artery dilatation	5 (31)	1 (2)	.004
Pericardial effusion	1 (7)	0	.28
Perivascular brightness of the coronary arteries	0	0	1
CAA	2 (13)	4 (9)	.66
Small (< 5 mm)	0	2	
Median (5–8 mm)	1	2	
Giant (> 8 mm)	1	0	
Long-term	3 (19)	2 (5)	.12
Coronary artery dilatation	2	0	
CAA	1	2	
Small (< 5 mm)	1	2	
Median (5–8 mm)	0	0	

Sole p-values < 0.05 are in boldfaced and p-values = 0.05 are not in boldfaced

After 6 weeks, the echocardiogram was also more frequently abnormal among resistant patients than responders (22% vs 7% respectively, *p* = 0.01). After 1 year, there was a trend for abnormal findings among the resistant group (9% vs 3%, *p* = 0.10): the only CAA of medium size was found in a patient resistant to IV Ig.

### Severity scores: distribution, sensitivity and specificity

There was no significant difference in the distribution of Kobayashi, Sano and Egami severity scores between the Ig- responder and resistant groups. Within the resistant group, the proportion of patients classified as severe was 22% for Kobayashi, 24% for Egami and 33% for Sano; likewise, the proportions within the group that responded to IV Ig and classified as severe was 22% for Kobayashi, 18% for Egami and 27% for Sano (Table 1). Sensitivity and specificity for the resistance to IV Ig were 22 and 78% for Kobayashi, 24 and 82% for Egami and 33 and 73% for Sano, respectively. Sensitivity and specificity for the prediction of the development of secondary coronary artery lesion were 17 and 77% for Kobayashi, 0 and 80% for Egami and 25 and 71% for Sano, respectively.

## Discussion

We retrospectively studied 157 children with KD in an observational and monocentric study. The monocentric nature of the study allowed a good coherence for the data with regard to diagnosis, treatment and evolution, thus limiting selection bias and optimizing the power in our cohort. The sex ratio (1.4), the proportion of children under 5 years old (85%), the most and least frequent clinical feature upon diagnosis (skin rash and lymphadenopathy), the proportion of resistance after the first Ig infusion (28%) and the presence of CAA in 7/157 (4%) patients after 1 year were consistent with other published studies [1–4, 28]. Similarly, treatment was also concordant with the standard of care of many other countries (2 mg/kg of IV Ig +/− corticosteroids for resistant patients) [29]. Our population of children with KD was thus comparable to those described in other developed countries.

In our study, IV Ig resistant patients had significantly more cardiac involvement at week 6, and a statistically non-significant trend to more frequent cardiac involvement at 12 months of evolution. Consistent with previous studies, resistant patients had a significantly lower decline of CRP after 48 h compared to responders [30]. It is notable that the modest size of our sample and missing data of the long-term follow-up for four patients (two in the immunoglobulin responder group and two in the resistant group) may have contributed to the fact the *P* value did not reach statistical significance at 12 months. These data are in agreement with data from the literature showing a link between IV Ig resistance and poor cardiac outcome [10, 12, 13].

We observed cardiac abnormalities in the first echocardiogram (performed at a median of 6 days after the onset of fever) in 31% of patients and in 11% of the echocardiograms performed at 6 weeks after disease onset. These two proportions were higher than in the literature [1, 13, 28]. This may be explained by the fact that we did not only consider coronary dilatations and aneurysms but all other abnormal echocardiographic findings, in particular perivascular brightness of the coronary arteries. In this context it is important to mention that coronary abnormalities may also be observed in diseases other than KD [31].

The available risk scoring systems from Japan (Kobayashi, Egami and Sano) were poor predictors of immunoglobulin resistance, with sensitivities below 33% and specificities below 81%. Our study population was of mixed origins (Sub-Saharan Africa, Europe, Northern Africa, Asia, the Caribbean and the Middle East). This heterogeneity reflects the reality of ethnic diversity in European countries. Similarly, other studies in mixed populations, for example a study conducted in a North American cohort (63% White, 17% Black, 17% Hispanic and 10% Asian) also found a low sensitivity (33–42%) of Japanese risk scores for predicting IV Ig resistance [21]. Thus, our work confirms the poor performance of available risk scoring systems in a European population, and emphasizes the need for alternative methods to identify severe disease.

Our study had several limitations. The monocentric design allowed for homogeneity of echocardiographic analysis and patient management, but limited its external validation. The proportion of patients with incomplete KD was higher than in other cohorts in the literature, although our results were similar in patients with complete or incomplete KD. Finally, the size of our cohort did not allow us to analyze the individual predictive value of each specific cardiac abnormality; subsequent studies using larger cohorts are required to address this point.

Though the definition of perivascular brightness of the coronaries was consensual among all cardiologists of our hospital, it remains a rather subjective criterion. It is important to mention that perivascular brightness can also be observed in the context of other diseases, such as septic shock. A recent study showed that baseline zMax ≥2.0 in children with Kawasaki disease is predictive for later development of CAA [32]. Together these studies strengthen the idea that early initial echocardiogram findings may have a crucial role for predicting a complicated disease evolution [27, 32, 33]. Unfortunately, in our retrospective study, z-scores were available only for more recently diagnosed patients, and we were therefore not able to analyze the predictive value of z-scores in our study population.

The most striking result of our study was that the presence of any cardiac abnormality in the early initial echocardiogram was strongly associated with resistance to IV Ig (*p* = 0.002), and development of coronary artery lesions within the first 6 weeks of the disease (*p* = 0.01). Additionally, all children with cardiac involvement at 12 months already had cardiac involvement at the first echocardiogram, and no patient with a normal initial echocardiogram showed persistent coronary abnormality after 1 year. Our results suggest that the presence of perivascular brightness of the coronary arteries and/or transient mild coronary artery dilatation, and/or pericardial effusion at the initial echocardiogram, should be taken into consideration for early identification of patients at high risk of a complicated disease evolution. Larger scale studies and/or prospective studies are now required to confirm or build upon these findings.

## Conclusions

Our study suggests that in a population of mixed ethnic backgrounds, the presence of any abnormality in the initial echocardiogram should be taken into consideration to identify patients at high risk for severe Kawasaki disease. Future studies should assess the value of using subjective echocardiography findings, in addition to standardized measurements (z-scores), for risk stratification purposes, especially with regards to patients with normal z-scores at the initial echocardiogram.

## Abbreviations

CAA: Coronary artery aneurysms
IV Ig: Intravenous immunoglobulin
KD: Kawasaki disease
